# Crowdsourcing and machine learning contests in Parkinson's disease research - when do they work?

**DOI:** 10.1177/1877718X261452116

**Published:** 2026-07-23

**Authors:** Leslie C Kirsch, Jeffrey M Hausdorff, Victoria J Dardov

**Affiliations:** 1 460644 The Michael J. Fox Foundation for Parkinson's Research, New York, USA; 2 Center for the Study of Movement, Cognition and Mobility, Neurological Institute, Tel Aviv Medical Center, Tel Aviv, Israel; 3 Sagol School of Neuroscience, Tel Aviv University, Tel Aviv, Israel; 4 Department of Physical Therapy, Gray Faculty of Medical & Health Sciences, Tel Aviv University, Tel Aviv, Israel; 5 Department of Orthopedic Surgery and Rush Alzheimer's Disease Center, Rush University Medical Center, Chicago, IL USA; 6 Technome.io, Herndon, USA

**Keywords:** Parkinson's disease, crowdsourcing, machine learning contests, collaborative science, open science

## Abstract

**Objective:**

To review the application of crowdsourcing and machine learning contests in Parkinson's disease (PD) research, identify best practices for successful implementation, and highlight future opportunities.

**Methods:**

This paper analyzes the landscape of crowdsourcing in PD research through a literature survey and a comparative case study of two major machine learning contests: the MJFF Freezing of Gait (FOG) Challenge and the AMP PD Proteomics Challenge. We also describe a taxonomy of crowdsourcing projects and a framework of success characteristics for machine learning contest design.

**Results:**

The analysis of previous crowdsourcing and machine learning contests revealed that contest success is highly dependent on specific design factors. The FOG challenge, which addressed a “solvable but not yet solved” problem with a suitable scoring metric, successfully produced a high-performing algorithm with real-world clinical value. In contrast, the Proteomics challenge did not yield biologically meaningful results, as winning models bypassed the core proteomic data, highlighting issues of data signal and metric selection. The review also identified underutilized crowdsourcing approaches in PD research, including gamification and community-based open-source development.

**Conclusions:**

Machine learning contests offer a powerful, open-science-aligned method to address complex problems in PD. Success requires careful design, particularly a solvable problem and an appropriate scoring metric. There is significant potential to expand the use of diverse crowdsourcing techniques to accelerate progress in PD research and clinical care.

## Plain language summary

This review explores the use of crowdsourcing techniques in the Parkinson's disease (PD) research ecosystem, with a focus on machine learning contests. We highlight best practices in designing crowdsourcing programs for successful research outcomes and provide an overview of opportunities for the PD research community.

## Introduction

Crowdsourcing is a distributed approach to problem solving, commonly defined as “the act of taking a job traditionally performed by a designated agent (usually an employee) and outsourcing it to an undefined, generally large group of people in the form of an open call”.^1^ Its steps include (1) an organizer identifying a problem to be solved, (2) the problem being broadcast online, (3) a crowd solving the problem, and (4) the organizer selecting a single or aggregated solution.^2^

Crowdsourcing approaches provide a variety of potential benefits when applied in a research context.^3^ It is an opportunity to draw interdisciplinary insight from collaborators and communities that would otherwise not engage with the research problem, e.g., particle physicists solving biochemistry challenges.^4^ It offers a distributed model for performing research work that can exponentially increase available resources, and provide a mechanism for persons with lived experience to contribute. From an open science perspective, it almost always requires intellectual work to be performed publicly, since the problem must be described for the crowd, and the crowd must describe their solutions for the community.

It also brings significant challenges, particularly in design and administration. It is not easy to design a *successful* crowdsourcing scenario, and even if the design is strong, maintenance activities are necessary to keep the crowd activated and driving toward the intended research purpose. The openness that attracts a variety of perspectives may discourage some participants, particularly in a biomedical context where the intellectual property stakes may be high. Biomedical crowdsourcing also has limitations due to ethical and privacy considerations; organizers must carefully consider issues of human subjects research implications. Finally for biomedical research, since participants in crowdsourcing are not necessarily subject matter experts, additional work is often necessary for organizers to ensure that the crowd's solutions are translated and vetted for the intended disciplinary audience.

**Figure 1.**
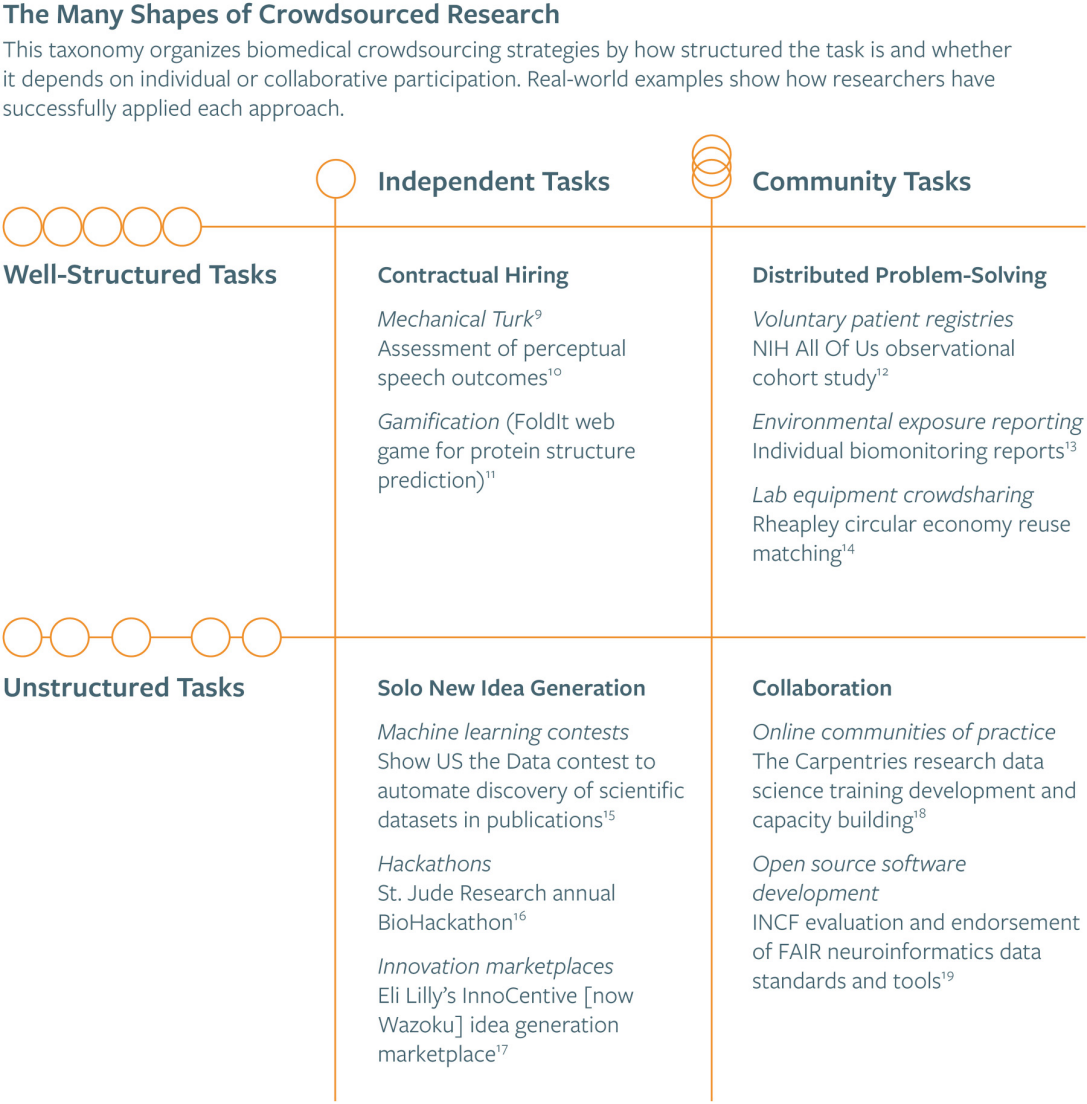
Crowdsourcing taxonomy with example biomedical research use cases.^9–19^

While machine learning competitions such as the Netflix Prize ($1 million won by AT&T Labs’ scientists),^5^ The Critical Assessment of Structural Prediction (won in 2020 by Google's AlphaFold 2),^6^ or the ALS Treatment Prize ($1 million won by the Institut de Myologie)^7^ are commonly provided as examples of crowdsourcing, there are many more tasks which fit the label. Crowdsourcing activities can be categorized by their degree of task structure, degree of participant independence, and level of resource required for participation.^8^ Structured tasks have a known solution, but it has not yet been executed fully; unstructured tasks have a solution space but the “best” answer is not known. Independent tasks are executed by individuals and aggregated, community tasks require coordination and teamwork. Figure 1 provides a taxonomy with examples of historic biomedical crowdsourcing projects.

**Table 1.** Machine learning contest platform features relevant to partner choice.^a^

Category	Characteristic	Description
Actionability	Code submission	Submission of code to take factors beyond accuracy into consideration in evaluating results, e.g., execution speed, resource consumption.
Actionability	Custom metrics	Ability to use a custom metric beyond the platform's standard offerings that is more relevant to the research question (which may incur a fee)
Actionability	Staged challenges	Ability to split a complex challenge into multiple phases to enable competitors to transfer knowledge of best solutions and continue working.
Actionability	Human evaluation	For challenges where effectiveness of results requires a human in the loop (e.g., a dialogue output), ability to connect to a human evaluation platform such as Mechanical Turk.
Actionability	Reinforcement learning evaluation	Since real-world computational environments are diverse, ability to assess results using repeated runs in randomized environments.
Community Engagement	Community activity	The degree to which the platforms offer an already engaged active community with relevant expertise.
Community Engagement	Run for free	Ability to join the competition without participants having to pay for the compute resources necessary to offer solutions
Open Science	Code reproducibility	Ability for participants to share and discuss their code with other competitors, whether through codesharing native to the platform or through a linked code repository (e.g., GitHub)
Open Science	Private challenge evaluation	For challenges involving restricted access or otherwise sensitive data, the ability to evaluate solutions using the organizer's own secure environments, rather than the partner or competitor's
Open Science	Open source	Whether the platform itself runs using open-source technologies.

^a^ Adapted from Rousseau and Ustyuzhanin (2020).

### Machine learning contests

Machine learning contests are online prize-based competitions where thousands of problem solvers can submit solutions to a specific computational challenge in the hopes of deriving the “best” solution amongst competitors and taking home a financial or non-financial reward.^20,21^ There has been a significant uptick in machine learning contests at major hosting platforms in the past three years, with industry analysts reporting an increase from 2022's estimated prize pool of US$5 million across 210 competitions^22^ increasing in 2024 to US$22 million across more than 400 competitions.^23^ The most popular online machine learning contest platform - Kaggle - has a community of over 24 million registered users, with 362 recognized as “grandmasters” based on their historic performance across competitions.^24^

Deep learning is a subset of machine learning. There are three main classifications of deep learning: supervised, semi-supervised and unsupervised. In this article, we will focus on supervised and unsupervised.^25^ Regardless of the specific machine learning technique, supervised learning uses labeled data for teaching or training the algorithm. This allows for generation of output using previous knowledge. Unsupervised learning utilizes unlabeled data to find structures or relationships in the data without explicit instructions. Most machine learning contests are solved through the use of supervised learning.

Machine learning contest organizers provide participants with a defined problem and supply the necessary data for a solution. Since the solutions will involve a machine learning algorithm, organizers must split their data into three parts - a training, validation, and test dataset.^26^ The training data is immediately made available to competitors to use in developing their solutions. Validation data is hidden from competitors, but they may submit their draft solutions to be scored against it to receive a draft ranking; it is also commonly called the “public leaderboard” data. The test dataset is completely private to competitors and is used by the contest organizer and host for the final scoring decisions. Without a test dataset, organizers risk overfitting their solutions to the available data, and solutions may not perform adequately in the real world.

While both machine learning contests and hackathons are independent-unstructured crowdsourcing projects, machine learning contests are differentiated from hackathons by the use of an objective scoring criteria, though both may offer a prize to the winner. Hackathons are generally more open-ended, resource-intensive for participants, and use subjective scoring; participants are commonly asking and answering unique questions and answers related to a domain problem. From a design perspective, a successful machine learning contest is measured first and foremost by the success of the solution, i.e., the performance improvement of the winning algorithm over established standards. For instance, the 2012 Antibody Sequencing Algorithm Challenge contest on TopCoder reduced time to complete a gene sequence annotation task by more than 96% over then-standard tools such as MegaBLAST, with just $6000 in prize money on offer and a 2-week window for 122 competing teams to submit.^27^ Organizers may have additional goals in mind, and the health of a competition in progress is often tracked by the unique or team participant count, total submissions/attempts, demographic and geographic diversity of competitors, etc. As examples, the Alzheimer's Disease Data Initiative's inaugural data challenge sought to promote use of their newly released Alzheimer's Disease Workbench,^28^ whereas the National Institute on Aging's PREPARE data challenge to predict ADRD allocated a third of its prize pool to the curation and contribution of high-quality datasets.^29^

Machine learning contests have many features which align strongly with open science principles. To be eligible for prizes, competitors must share the complete details of their algorithm with the organizers, and organizers can further require that all solutions be provided for community use under an open-source license such as CC-BY. However, machine learning contests also require *private* data to succeed; the organizers must have a test data set to evaluate responses which are not accessible to competitors. Therefore, contests cannot simply repurpose existing open research data because it is discoverable to unethical competitors. Most contest organizers lack the infrastructure to run an event on their own and partner with a specialized platform, such as Kaggle, Synapse, HackerEarth, or others. These partners are critical to the success of the contest, but their practices may not be ideally aligned with sponsors’ policies or practices related to open science and sponsors may need to carefully consider tradeoffs. For instance, while Kaggle includes open code sharing and preserves data, it does not assign research-friendly persistent identifiers to key outputs such as digital object identifiers or research resource identifiers. These partner practices change over time and may or may not offer flexibility, so sponsors must review options during the planning process. Prior research has suggested that sponsors consider a variety of platform characteristics in balancing priorities such as actionability of results, community engagement, and open science policies, as shown in Table 1.^30^

Not every machine learning contest is successful, but contest administrators and researchers have identified characteristics which influence success, as shown in Table 2.

**Table 2.** Successful machine learning contest characteristics.

Category	Characteristic	Description
Design	Clear question	Clearly defined and narrow question, meaningfully explained for an interdisciplinary audience.^31^
Design	Desirable to participants	Competition centers on solving a real-world problem that will have a clear benefit, explained understandably for participants to want to contribute answers.^32^
Design	Solvable but not yet solved	Organizers have good reason to believe there is signal in the noise, but not already know the answer - there should be space for competitors to beat the current “gold standard”
Design	Good scoring metric	An objective scoring method can be assigned which is meaningful to assess answers and precise enough to differentiate to score a close competition.^33^
Data	Enough data	Adequate volume of data to meet best practices in training-test-validation (e.g., 80/10/10) splits
Data	Quality data	Unambiguous “ground truth” for training data
Data	Robust infrastructure	Technological infrastructure underpinning the contest is able to support the data science needs of participants, whether through a centrally managed hosting platform or through distributed resources supplied by participants.^34^
Culture	Trust in the host and organizer	Trust in the hosting platform and contest organizer to manage a fair and equitable competition with clear rules and resource assignment.
Culture	Useful leaderboard	Clarity for competitors on their relative performance to encourage effective iteration and improvement.^35^
Culture	Effective moderation	Effective moderation to respond to competitors, clarify, adjust, and provide encouragement.^36^

In the specific context of PD, contest organizers should be aware that meeting the success characteristics for “enough data” and “quality data” can be a complex issue. Depending on the nature of the question to be addressed by the machine learning challenge, a random training-test-validation split may not be appropriate. For instance, in predicting a question around progression, data from early repeated measures may be a proxy for later measures (e.g., imaging scans showing dopaminergic neuron loss). To prevent a model from learning a specific participant's characteristics, it is also necessary to split longitudinal data by participant, time or clinical site rather than using a random split. For cross-sectional predictions, many encoded values could inadvertently serve as diagnostic proxies, inadvertently acting as a form of leakage. Data leakage refers to a situation where the model has access to information during training that would not be available in real-world use (e.g., future data or proxy variables). This allows the model to “cheat” by using data that would not actually be available at the time of prediction in the real world. As noted above, it is also very important to choose a “meaningful” scoring method; poorly chosen scoring methods can reward algorithms which prove extremely successful at predicting scientifically unimportant information (e.g., a prediction of genetic risk may actually be predicting clinical site). While experts in machine learning model design are often available from partner vendors, organizers should be prepared to provide subject matter expertise about PD to ensure effective design. Further, during design, organizers should consider the data curation necessary for the data to meet quality standards specific to the research question. For instance, individuals may be labeled as “healthy controls” at the time of recruitment for a study who were ultimately found to have PD; mislabeled training data such as this will negatively impact the quality of results. Similarly, the nature and quantity of data may lend itself toward overfitting, where the model works extremely well, but only for the contest dataset, e.g., a model built from only European ancestry data may not work as well for African ancestry data. If a second, external validation set is available that can also be included in the contest, e.g., collected in a different cohort or different circumstances, that can significantly help for evaluating and potentially demonstrating the generalizability and robustness of the best model, avoiding the overfitting limitations.

## Crowdsourcing in PD research

### PD-related examples across the crowdsourcing spectrum

**Figure 2.**
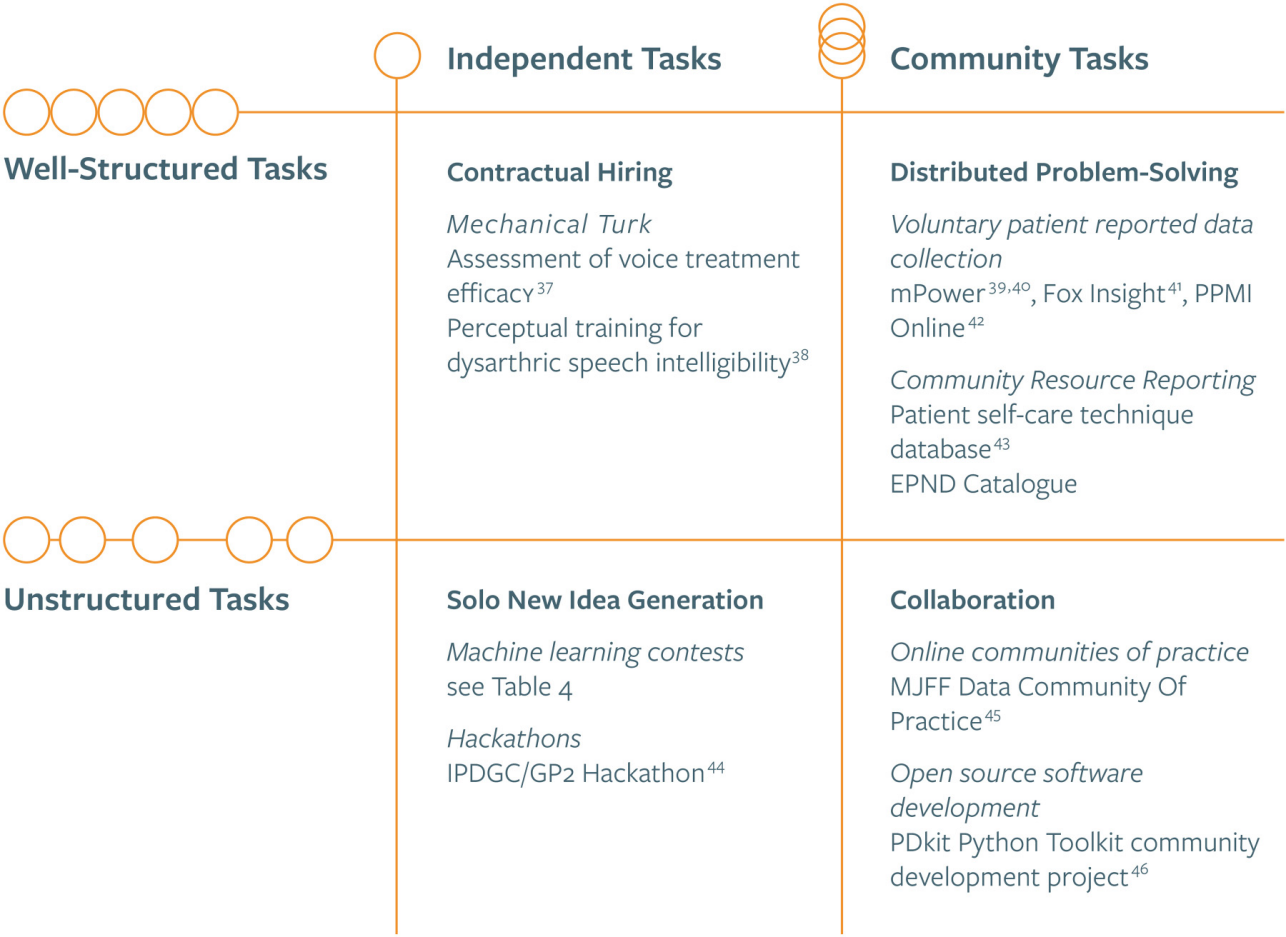
PD crowdsourced research projects.^37–46^

The PD research community has used crowdsourcing approaches to tackle a variety of problems, as shown in Figure 2.

### Machine learning contests in PD research

With the rise of generative AI and increasing interest in applying advanced computational techniques to biomedical problems, the Parkinson's disease research community has seen multiple machine learning contests in the past ten years, as shown in Table 3.

**Table 3.** Recent machine learning contests tackling Parkinson's disease-related topics.

Challenge (year)	Platform (USD total prize)	Data type	Participants (Teams)	Submissions	Outcome
FDA FoG (2024)^47^	PrecisionFDA ($0)	Time-series (digital sensor)	15 (7)	undisclosed	ongoing
MJFF FoG^48^ (2023)	Kaggle ($100,000)	Time-series (digital sensor)	1688 (1379)	28,132	Well-performing algorithm with real-world value
AMP PD Proteomics (2023)^49^	Kaggle ($60,000)	Tabular (proteomics)	2197 (1805)	40,764	No scientifically meaningful solutions identified
BEAT PD (2020)^50^	Synapse ($25,000)	Time-series (digital sensor)	Undisclosed (42)	38	Demonstrated proof-of-concept but not real-world value
PDDB DREAM (2017)^51^	Synapse	Time-series (digital sensor)	Undisclosed (23)	39	Limited improvement on base models

### Designing a successful machine learning contest - lessons from the AMP PD proteomics challenge and the MJFF freezing of gait challenge

**AMP PD Proteomics Challenge:** To discover association between the protein and clinical data, AMP PD sponsored a Kaggle challenge where the goal was to develop a model trained on data of protein and peptide levels over time in subjects with Parkinson's disease versus normal age-matched control subjects. The ultimate goal of this competition was to predict MDS-UPDRS scores utilizing proteomics data and determine if any proteins or peptides could be associated with MDS-UPDRS changes. The Movement Disorder Society-Sponsored Revision of the Unified Parkinson's Disease Rating Scale (MDS-UPDRS) is a comprehensive assessment of both motor and non-motor symptoms associated with Parkinson's Disease.^52^ This clinical measure is correlated with severity of disease and takes many facets, like mood activities of daily living and motor function, into account for calculations. DIA (Data Independent Acquisition) proteomics aims to measure as much of the proteome as possible in a given sample.^53^ AMP PD performed DIA proteomics analysis and set aside a subset of the CSF data specifically for the Kaggle data challenge. Both protein and peptide data were provided for this challenge along with MDS-UPDRS and additional clinical and visit data. No demographic data or other cohort stratification data was provided, in order to protect participant privacy.

1805 teams from all over the world participated in this challenge, with 2197 participants and 40,764 submissions. SMAPE+1 was used as the scoring metric to determine how well the submitted models performed. The community within this data challenge was supportive of each other throughout the competition, with competitors often answering questions that were posted.^54^ The winning team's solution focused solely on clinical data, ignoring the provided CSF (protein/peptide) data.^55^ Their model used features such as visit month, forecast horizon, target prediction month, and other clinical indicators. Despite efforts to find a signal in the blood test data, they concluded it did not provide significant information and trained their final models only on clinical and supplementary data. The finding that visit number was predictive of MDS-UPDRS is not surprising as most patients progress in disease severity as they continue their visits and is not considered a scientifically important predictor.

Overall, participants who did not use the CSF protein or peptide data generally had lower SMAPE+1 and therefore scored higher in the competition rankings.^56^ While the organizers did adjust the scoring metric from SMAPE to SMAPE+1 mid-contest in response to preliminary leaderboard results, it did not change the dominance of visit metadata-driven predictions. This led to lessons learned, including the need for explicit directions in future challenges and questioning the suitability of the SMAPE+1 scoring metric for this specific challenge. Some participants noted that low scores on MDS-UPDRS may not be adequately sensitive measures of disease progression for this purpose, adding randomness in the context of SMAPE-based scoring.

The lack of success in this data challenge highlights how even thoughtfully designed challenges might not yield biologically relevant solutions. There are also outstanding questions about why using protein/peptide data consistently resulted in lower scores and whether providing a larger dataset or both CSF and Plasma data would have changed the outcome. In addition, perhaps the right questions were not being asked of the data - the question of whether proteins or peptides correlated with MDS-UPDRS progression might not have been the right one for this case. Perhaps a question asking participants to determine peptide level changes while protein levels stayed the same and see if that is associated with disease progression could have led to different outcomes, or one using engineered functional annotations of the data. The subset of CSF proteomics data provided as the training data set in the Kaggle challenge is now available to registered AMP PD users as part of the full data set through AMP PD.^57^

**MJFF Freezing Of Gait (FOG) Challenge:** Freezing of gait (FOG) is a sudden, episodic, and unpredictable disruption in walking that affects a substantial subset of individuals with Parkinson's disease (PD).^58^ During a FOG episode, patients describe an inability to initiate or continue walking, often reporting that their feet feel inexplicably “glued” to the floor. Forward progression is halted, sometimes for a fraction of a second, sometimes for much longer, despite the patient's intention to move. FOG is associated with a range of adverse outcomes, including a significantly increased risk of falls, diminished health-related quality of life, and loss of independence.^59,60^ In severe cases, patients may become wheelchair-dependent. Despite its major clinical relevance, FOG remains poorly understood, and effective treatments are lacking.^61^

A major obstacle in advancing FOG research and clinical care is the difficulty in objectively and consistently assessing the severity of these episodes.^62^ Many prior studies have applied signal processing and machine learning techniques to data captured from wearable sensors—such as accelerometers—to detect and quantify FOG. While these efforts have yielded promising results, progress in the field has been hindered by two major limitations: the predominance of small, single-center datasets that limit generalizability, and the absence of a publicly available, standardized, relatively large dataset that would enable consistent benchmarking and comparison of algorithms.

To address these challenges, the Michael J. Fox Foundation (MJFF) sponsored a machine learning competition hosted on the Kaggle platform.^63^ The competition aimed to stimulate innovation by crowdsourcing algorithm development from the broader data science community. It was designed to attract experts in AI who were not necessarily familiar with PD. The challenge was announced both to the Kaggle community—comprising over 8 million users—and to the PD research field. Submissions were open for 3 months.

A relatively large, curated dataset was released as part of the challenge. It included raw acceleration signals captured from wearable sensors, paired with expert annotations of FOG events derived from video reviews. The contest site also provided a clear description of the evaluation metrics and the goals for automated, objective FOG detection. A total prize pool of $100,000 was awarded to the top five performing teams based on the MAPE metric. A leader board was posted on the contest website, giving updates throughout the 3 months on the top scoring algorithms (based only on the public dataset; the actual scoring was based on a private dataset).

In total, 1379 teams from 83 countries submitted 24,862 model solutions. During the 3 months, helpful interactions and discussions were made on the Kaggle platform, allowing participants to learn from each other in a spirit of comradery. Notably, many of the winning teams had no prior experience in PD research. Despite this, their solutions achieved high accuracy, specificity, and precision in detecting FOG episodes, demonstrating strong agreement with expert annotations. When applied to continuous 24/7 monitoring data, these models revealed previously uncharacterized patterns of FOG during daily living.

This successful initiative highlights the power of crowdsourced machine learning competitions in accelerating progress on complex biomedical problems. It provides a compelling demonstration of how open data, interdisciplinary collaboration, and global engagement can drive advances in the objective measurement and understanding of disabling symptoms in PD.

**Figure 3.**
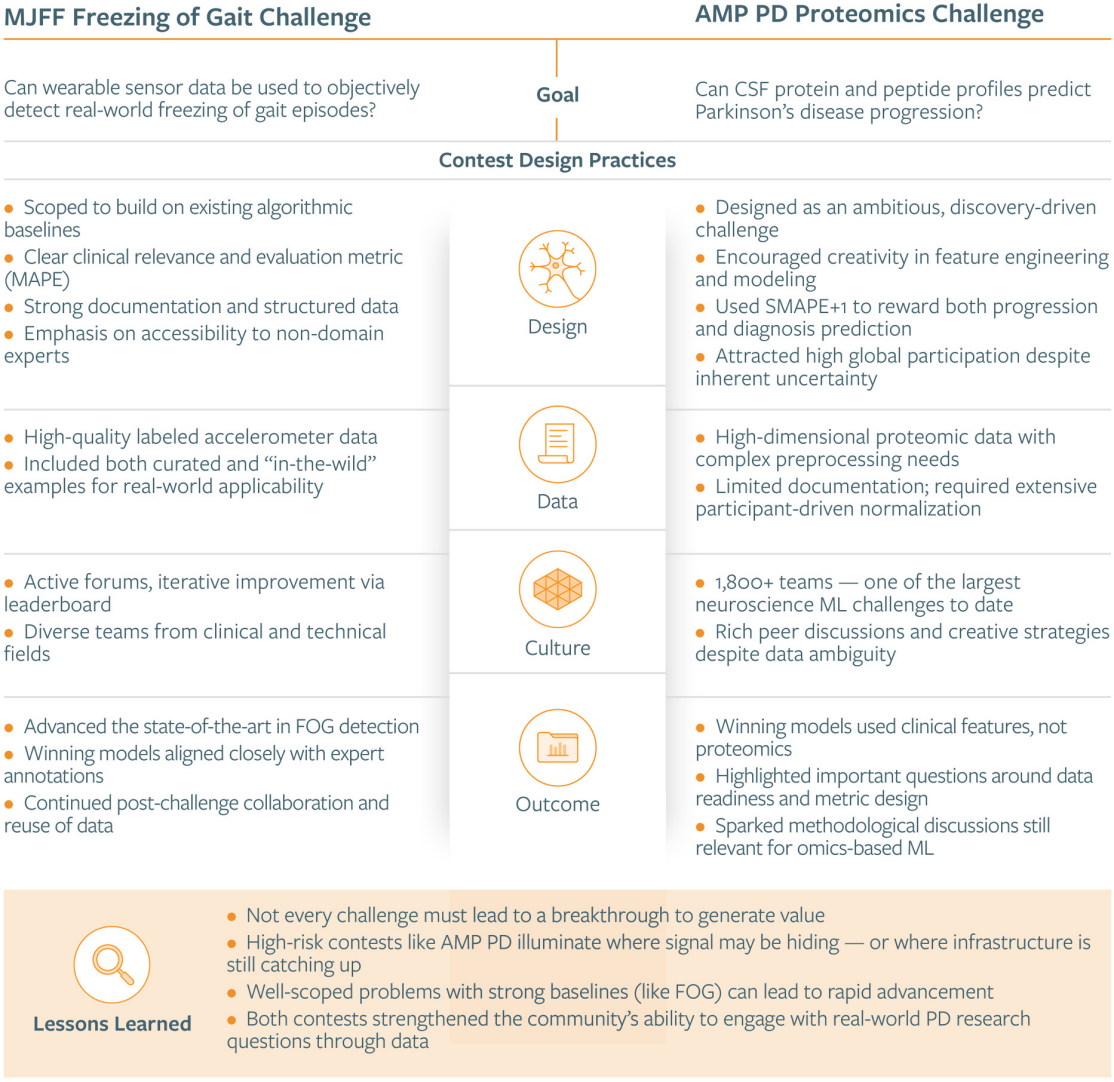
Comparison of two major PD-focused machine learning contests.

**Lessons Learned:** While both challenges were designed keeping the ten success characteristics from Table 1 in mind and both successfully attracted a large number of diverse competitors from Kaggle's existing community, only one resulted in scientifically and practically actionable advancement in the state of the field, as described above. In a post-hoc review, this difference in higher success for the FOG challenge can be primarily attributed to the relative strength of design on two specific design characteristics: a “solvable but not yet solved” problem and a “good scoring metric.” Figure 3 below shows a comparison of the experiences under these two machine learning contests.

Specifically, in the FOG challenge, organizers knew that algorithms already existed to predict FOG outcomes from the provided input data, but algorithmic performance had room for improvement on both precision and sensitivity. Crowdsourcing provided a beneficial approach by quickly applying significant resources in the form of technical algorithmic expertise by a large number of users and resulted in a useful improved ensemble model. In contrast, for the AMP PD Proteomics challenge, a priori evidence did not exist to clearly demonstrate known predictive value for the input data. Rather, the challenge was known to be low-likelihood, high-reward - if predictive power was found, the individually predictive features would be incredibly scientifically meaningful, but there was no guarantee of success. While these factors likely contributed to the differing outcomes between the two challenges, we acknowledge that this interpretation is partly post hoc. Other elements including differences in data modality, signal-to-noise characteristics, and the degree to which the tasks aligned with the strengths of the participating data science community may also have influenced the results.

Further, in the FOG challenge, the scoring metric of MAPE was ultimately a good match for the nature of the input data and the desired outcome. MAPE is robust to the inclusion of meaningful zero values, and its weakness is that it penalizes overprediction more than underprediction. However, with a significant count of challenge entries, this weakness is mitigated by volume, wherein the winning solutions are essentially differentiated by strong performance in balancing positive and negative errors. While the winning solution was an improvement on the state-of-the-art, its low rate of successful prediction for less common forms of FOG may have been driven in part by the scoring metric choice. In the AMP PD challenge, the suitability of the scoring metric of SMAPE – later adjusted to SMAPE+1 - was a point of significant discussion among participants.^64^ It was selected by organizers with the intent that it would theoretically place some weight on participants’ ability to predict a participant's PD status. However, since SMAPE+1 still weights toward low relative magnitude differences, high rates of low UPDRS scores in the actual dataset overshadowed every other possible predictor in actual scoring. The winning algorithms therefore achieved high scores by ignoring the proteomics data and optimizing toward the scoring metric in ways that did not align with biological objectives. Results suggest that if there is predictive power in the proteomics data, it would require a different dataset to pull the signal from the noise.

## Opportunities and challenges for future crowdsourcing in PD research

In revisiting the crowdsourcing taxonomy, we note that certain quadrants are more heavily represented in PD research than others. There is certainly no shortage of highly successful projects tapping the community for well-structured tasks, particularly in the area of voluntary patient-reported outcome measures. However, there are fewer examples available of successful crowdsourcing projects that are (1) independent and well-structured or (2) community collaborations. Crowdsourcing projects in these categories have resulted in significant benefit in other arenas of biomedical research, such as the examples provided in Figure 1. PD researchers may specifically wish to consider the benefits of gamification of repetitive research problems or opportunities for community-based open-source software development projects.

Drawing from the examples of other biomedical specializations, some potential opportunities in crowdsourced research could include:

1. Community-developed open source hardware: With the popularity of personal 3D printers, it is increasingly feasible to crowdsource the design and construction of small hardware and durable tools via crowdsharing sites such as Thingiverse.^65^ A search for “parkinson's” shows a variety of small personal care and treatment tools, such as Brian Alldridge's Parkinson's-Friendly Pill Dispenser^66^; researchers may wish to consider tapping into this enthusiastic crowdsourcing community to help build tools that could help in the conduct of research.
2. Lab equipment crowdsharing: Open science initiatives have greatly enhanced the number of reusable research resources available to the PD research community, often in the form of output data, preclinical tools and models, code and software. Specialized equipment crowdsharing has shown some success in clinical care settings. While many PD research teams are incredibly collaborative and generous with resources at a partnership level, open science programs could consider whether additional opportunities exist for Rheaply-style fractional equipment sharing or reuse at a larger scale.
3. Community resource reporting: PD researchers are collecting data and biospecimens at an incredible rate, thanks to the passionate engagement of the patient community. While PD has embraced patient-reported information as a form of research data collection, there has been less activity focused on finding a scalable way to enable researcher-reported resource information on siloed data and biospecimens. Increasing visibility of available resources honors the immense contribution of the patient community by maximizing resource reuse. Funders and consortia such as Aligning Science Across Parkinson's, the European Platform for Neurodegenerative Diseases, and the Michael J. Fox Foundation for Parkinson's Research are positioned to enable the technology needed for effective researcher resource self-reporting, but the research community will have to embrace the crowdsourcing activity of reporting for success.

While there has been considerable interest in PD research in using unstructured independent tasks such as machine learning contests and hackathons for PD research, these crowdsourcing techniques could still be viewed as underutilized considering the state of the broader biomedical field. PD machine learning contests have thus far focused primarily on time-series sensor data, and the success of the recent FOG challenge is indebted to the learnings of earlier similar challenges. Time-series sensor data is particularly well-suited to machine learning contests, since many challenges in sensor data are knowably solvable, such as optimization and sensitivity, and these challenges require a relatively small number of participants to generate an adequate volume of relevant data. Other types of problems which may be good fits for future data challenges in PD may include:

1. Imaging/computer vision: Generative AI models and improvements in the field of computer vision provide opportunity for meaningfully reprocessing existing raw image data. Difficulties include locating an adequate volume of previously unreleased data and ensuring adequate de-identification for challenge purposes.
2. Voice data: Many fit-for-purpose voice data processing algorithms have been commercially and openly developed for general speech processing. While voice data can be a challenge for de-identification, like time-series sensor data, it also provides rich datasets in relatively small participant counts to tackle challenges such as predicting PD risk, providing early diagnosis, or detecting progression via changes in speech patterns. Necessary data can also be collected remotely, making prospective collection for challenge purposes more feasible.
3. EEG data: Machine learning algorithms have demonstrated some success in predicting PD diagnosis or cognitive impairment from EEG data, but studies have been characterized by inconsistencies in data preprocessing and model evaluation.^67^ ML contests on Kaggle tackling “solvable but not yet solved” and featuring large amounts of high-quality EEG data have demonstrated positive results in epilepsy research.^68^

We provide success criteria in Table 1 for organizers to consider when contemplating a machine learning contest for PD research. As with any research project, time spent on thoughtful design increases the likelihood of success. However, no matter how well designed, no machine learning contest is guaranteed to succeed - the contest will always have a “winner” but it may not be meaningful in advancing scientific understanding. As seen in the case examples here, there are myriad opportunities for unexpected outcomes, given that contest participants have an interest in creating a technically superior algorithm regardless of the science. Assuming that high quality data exists, machine learning contests are relatively inexpensive and can draw massive specialized resources to apply to a problem, so organizers with some risk tolerance may find it appealing to test even imperfect problems. From an open science perspective, there is also great benefit in having so many algorithm developers work out loud, so that future researchers can effectively build on their code.

For machine learning contests specifically, organizers with an interest in open science should strongly consider including *reproducibility* and *explainability* as prize criteria. An explainable machine learning model allows a user to understand what data elements contributed to the model's output; explainability is intended to provide information to determine trustworthiness of a model and provide quality control during its use.^69^ While it may be difficult to scientifically interpret the potentially high number of factors contributing to a transparent model's prediction, this transparency provides value to the PD research community. Prior research has shown that transparent algorithms perform as well as so-called “black box” models, and they would provide considerably greater practical value in PD research.^70^ See Table 4 for a summary of open science-friendly prize criteria concepts which organizers of PD machine learning challenges may wish to consider.

**Table 4.** Open science-friendly prize criteria concepts for PD machine learning challenges.

Concept	The question it answers	The goal
Transparency	"How was this built and what is inside?"	Accountability (Knowing who to blame or how to fix it).
Interpretability	"How does the machine think?"	Trust (Believing the internal logic makes sense).
Explainability	"Why did it make this specific choice?"	Justification (Proving a specific diagnosis or prediction).

Understanding why a model is successful can illuminate scientifically valuable information on potential targets and pathways, and provide avenues for future discovery or translational research. Model Cards are becoming the industry standard for providing transparency by documenting a model's strengths, limitations, and training biases. See Table 5 for an example for a hypothetical gait-based PD prediction algorithm.

**Table 5.** Sample model card: gait-based PD predictor.

Section	Content	Purpose
Model Details	Random Forest Classifier (v1.2). Trained on 7-day accelerometer data.	Algorithmic Transparency: Identifies the specific model type and version.
Intended Use	Screening for early-stage Parkinson's Disease in adults aged 65 + . Not for definitive diagnosis.	Process Transparency: Defines the scope and “safe” boundaries of the tool.
Factors	Age, Sex, Average Step Velocity, Gait Variability.	Interpretability: Lists the features, showing how “human-readable” the input is.
Metrics	AUC: 0.88; Sensitivity: 0.82; Specificity: 0.85.	Performance Transparency: Shows how the model actually performed on the test set.
Training Data	1200 subjects from Site A (Clinical setting).	Data Transparency: Reveals the source and potential “site effects."
External Validation	Tested on 300 subjects from Site B (Free-living environment).	Robustness: Proves the model wasn't just “leaking” information from Site A.
Explainability	SHAP values used to rank feature importance for each prediction.	Explainability: Describes the tool used to peek inside the “black box."

In general, crowdsourcing in its myriad forms has shown promise within the PD research community. Nonetheless, great opportunities remain to expand its usage. Crowdsourcing is inherently open science-friendly, since the crowd can only be effectively activated when knowledge is shared between organizers, facilitators, and individual contributors. Future crowdsourcing projects within PD research can take advantage of best practices described here to increase their likelihood of success and expand their impact on research and clinical care.
